# STROBE—Radiation Ulcer: An Overlooked Complication of Fluoroscopic Intervention: A Cross-Sectional Study

**DOI:** 10.1097/MD.0000000000002178

**Published:** 2015-12-07

**Authors:** Kai-Che Wei, Kuo-Chung Yang, Guang-Yuan Mar, Lee-Wei Chen, Chieh-Shan Wu, Chi-Cheng Lai, Wen-Hua Wang, Ping-Chin Lai

**Affiliations:** From the Department of Dermatology (K-CW, C-SW) and Department of Plastic and Reconstructive Surgery (K-CY, L-WC), Kaohsiung Veterans General Hospital, Kaohsiung, Taiwan; Faculty of Yuhing Junior College of Health Care and Management, Kaohsiung, Taiwan (K-CW); Department of Cardiology, Kaohsiung Veterans General Hospital, Kaohsiung, Taiwan (G-YM, C-CL, W-HW); National Yang-Ming University School of Medicine, Taipei, Taiwan (L-WC); and Department of Nephrology, Kidney Center, Chang Gung Memorial Hospital, Chang Gung School of Medicine, Chang Gung University, Linkou, Taiwan (P-CL).

## Abstract

With increasing numbers of percutaneous coronary intervention (PCI) and complex cardiac procedures, higher accumulated radiation dose in patient has been observed. We speculate cardiac catheter intervention induced radiation skin damage is no longer rare.

To study the incidence of cardiac fluoroscopic intervention induced radiation ulcer.

We retrospectively reviewed medical records of those who received cardiac fluoroscopic intervention in our hospital during 2012 to 2013 for any events of radiation ulcer. Only patients, whose clinical photos were available for reviewing, would be included for further evaluation. The diagnosis of radiation ulcers were made when there is a history of PCI with pictures proven skin ulcers, which presented typical characteristics of radiation injury.

Nine patients with radiation ulcer were identified and the incidence was 0.34% (9/2570) per practice and 0.42% (9/2124) per patient. Prolonged procedure time, cumulative multiple procedures, right coronary artery occlusion with chronic total occlusion, obesity, and diabetes are frequent characteristics. The onset interval between the first skin manifestation and the latest radiation exposure varied from 3 weeks to 3 months. The histopathology studies failed to make diagnosis correctly in 5 out of 6 patients. To make thing worse, skin biopsy exacerbated the preexisting radiation dermatitis. Notably, all radiation ulcers were refractory to conventional wound care. Surgical intervention was necessary to heal the wound.

Diagnosis of cardiac fluoroscopy intervention induced radiation skin damage is challenging and needs high index of clinical suspicion. Minimizing the radiation exposure by using new approaches is the most important way to prevent this complication. Patient education and a routine postprocedure dermatology follow up are mandatory in high-risk groups for both radiation skin damage and malignancies.

This is a retrospective study, thus the true incidence of radiation ulcer caused by cardiac fluoroscopic intervention could be higher.

## INTRODUCTION

Radiation ulcer is the most severe form of radiation dermatitis, which is the consequence of skin injury from exceeding cumulative radiation dose. Radiation ulcer has been thought to be rare but the recent publications showed that its incidence is on the rising.^1,2^ This is partly due to the broad application of interventional cardiology and percutaneous coronary interventions (PCI) increase noticeably in current medical practice. According to the registry, more than 1 million procedures of cardiac fluoroscopic interventions are done annually in the United States^3,4^ and an estimated 35,000 in Taiwan (data from the National Health Insurance Database of Taiwan). On top of this, with advancements of new stent implantation techniques, the numbers of more complex procedures increase significantly.^5^ These complex procedures inevitably lead higher radiation dose in each procedure.^6,7^ Repeated multiple procedures have also become more common; therefore, the lifelong cumulative radiation dose becomes much higher. Putting these together, it would not be a surprise if radiation dermatitis following PCI is no longer as rare as once it was thought.

## MATERIALS AND METHODS

This retrospective study complied with the guidelines of the Declaration of Helsinki and was approved by the Medical Ethics Committee of Veteran General Hospital, Kaohsiung, in Taiwan. Institutional Review Board approval was obtained, and because the study involved retrospective review of existing data, informed consent from the patients were not required. In addition, all individuals’ information was securely protected (by delinking identifying information from the main data set) and available to investigators only. Furthermore, all the data were analyzed anonymously. All primary data were collected according to Strengthening the Reporting of Observational Studies in Epidemiology guidelines.

We conducted this study by reviewing cases records of all patients who had received either PCI or electrophysiologic ablation (EPA) between 2012 and 2013 in our hospital. Only patients, whose clinical photos were available for reviewing, would be included for further evaluation. The diagnosis of radiation ulcers were made when there is a history of cardiac fluoroscopic intervention with pictures proven skin ulcers, which presented typical characteristics of radiation injury. Their demographic data, procedures received, approximate dosage of radiation, and skin lesion-related medical data were collected and analyzed and presented as mean ± SD. The incidence rate per patient is defined as radiation ulcer patients/total patients, while the incidence is defined as radiation ulcer patients/total procedures.

## RESULTS

From January 2012 to December 2013, 2570 times of percutaneous cardiac catheterizations (total 2124 patients, male/female ratio = 4.41, average age of male = 64.25 ± 13.56 years old, average age of female = 69.45 ± 9.59 years old) were performed in our hospital. These procedures included PCI (total 2454 procedures) and EPA (total 116 procedures). Among PCIs group, 238 cases received complex PCI for chronic total occlusion (CTO).

Nine cases with radiation skin ulcer were identified (Table 1). The incidence rate of radiation ulcer developed in 2124 patients, who had received cardiac fluoroscopic intervention during January 2012 to December 2013, was 0.34% per practice (9/2570) or 0.42% per patient (9/2124). Their mean age is 60.7 ± 14.4 years old with a range between 42 and 82 years old. All of them are male. Six of them were obese with BMI ≥ 27, while 7 were of diabetes mellitus. None had history of autoimmune disease. Eight patients received PCIs for coronary artery disease (CAD), whereas 1 patient received EPA for his accessory pathway. Among patients with CAD, 3 had triple vessel disease, and the rest had double vessel disease. Notably, all of them had occlusion of right coronary artery and 5 of them were with CTO. Percutaneous angiographic interventions had been performed at least 3 times in all patients within recent 7 years. The average accumulated fluoroscopy time (of all fluorosopic procedure within recent 7 years) for each patient was 379 ± 212 min (ranging from 166 to 801 min). If the fluoroscopy time is converted to entrance skin dose with radiation exposure rate 0.05 to 0.1 Gy/min, the estimated entrance skin dose was at least ranging from 8.3 to 40.1 Gy.

**TABLE 1:**
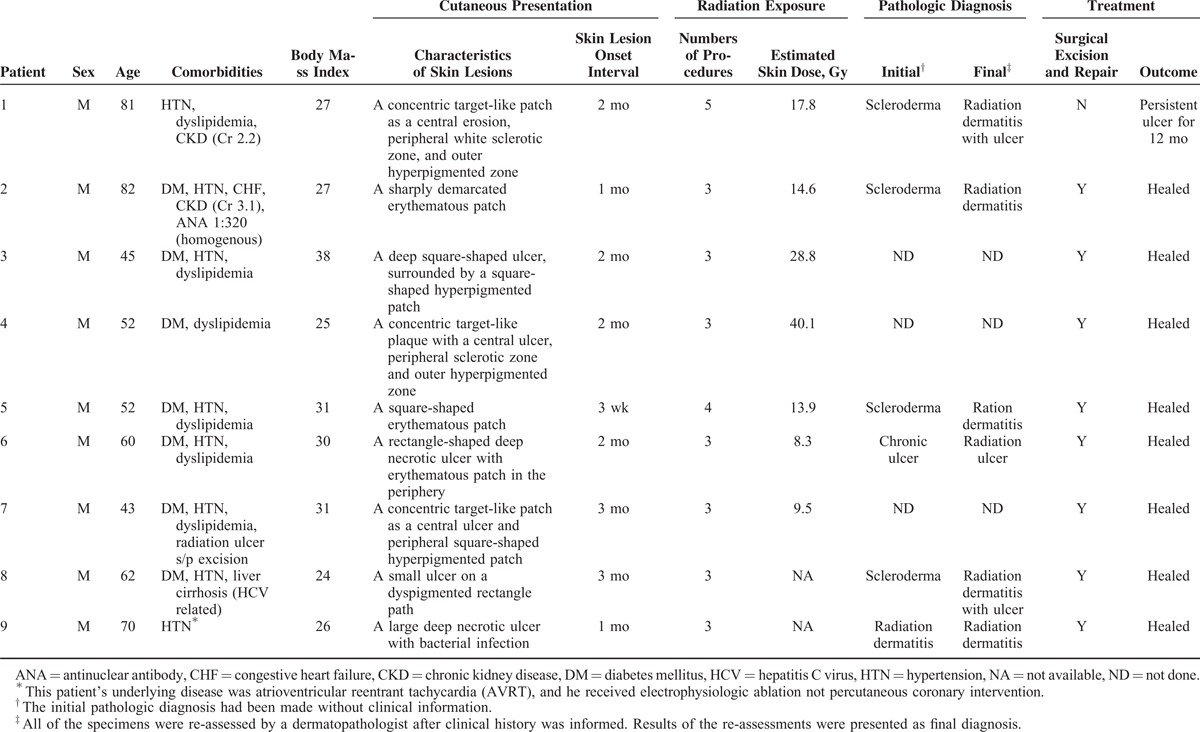
Demographic Data and Treatment Courses of Radiation Ulcer Patients

None of these patients had history of acute skin damage at the lesion site immediately after receiving fluoroscopic procedure. The interval between the onset of first skin manifestation and the latest radiation exposure ranged from 3 weeks to 3 months. Itch and pain were noted in all patients. None of them had been self-aware the connection between the previous cardiac fluoroscopic intervention and the development of the skin lesion at the first presentation to clinic.

**FIGURE 1:**
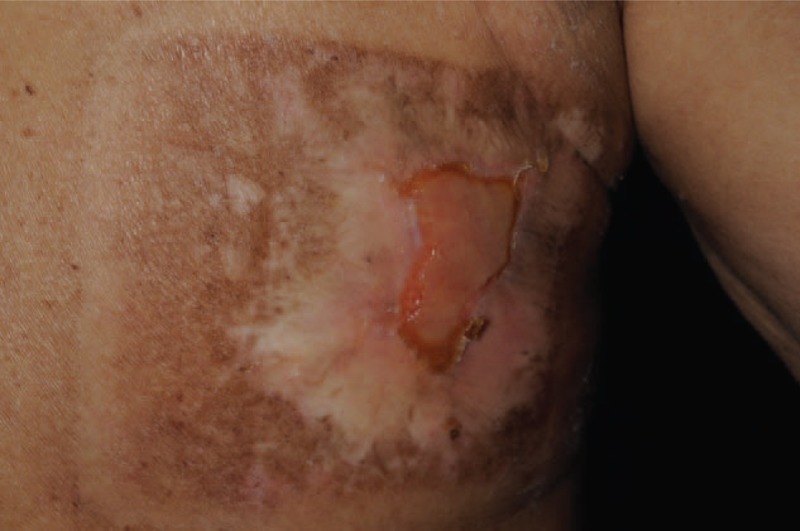
Typical presentation of cardiac fluoroscopy-induced radiation dermatitis before ulceration. An 81-year-old man presented with a painful sharply demarcated concentric target-like patch. A central erosion with a peripheral white sclerotic zone and an outer hyperpigmented “flame burn-like” zone.

**FIGURE 2:**
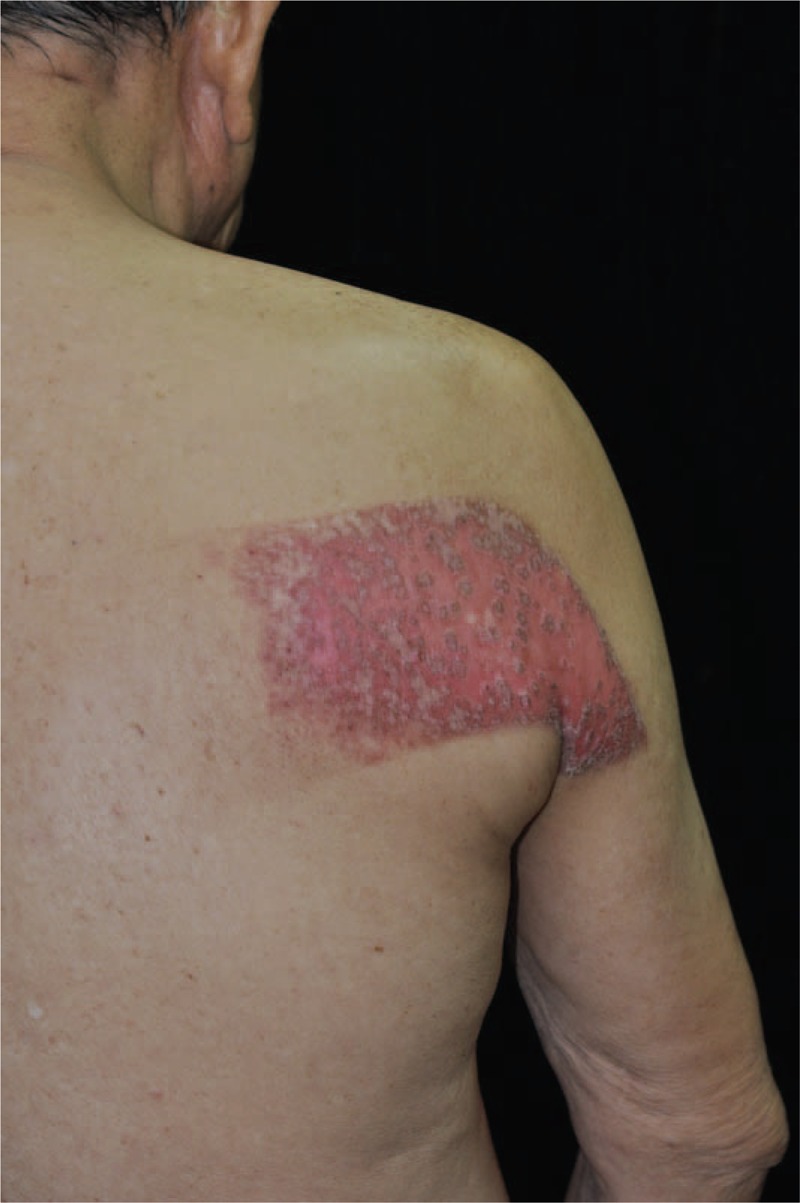
Radiation dermatitis mimicking contact dermatitis. An 82-year-old man presented with an itchy painful sharply demarcated rectangular erythematous patch with dry fish scale-like desquamation on right subscapula, auxiliary and inner arm. The initial clinical diagnosis was contact dermatitis.

**FIGURE 3:**
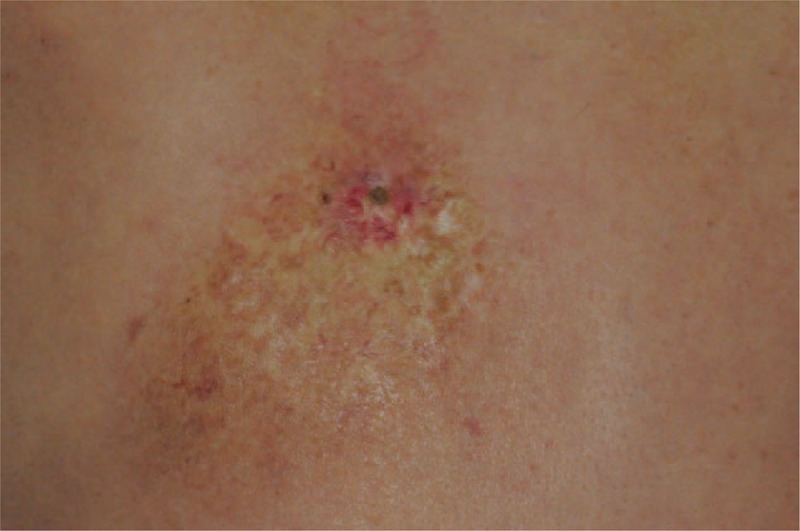
Radiation dermatitis mimicking scleroderma. A 62-year-old man presented an itchy painful bizarre-shaped atrophic telangiectatic plaque with a central small ulcer and heterogeneous color change on his mid back.

**FIGURE 4:**
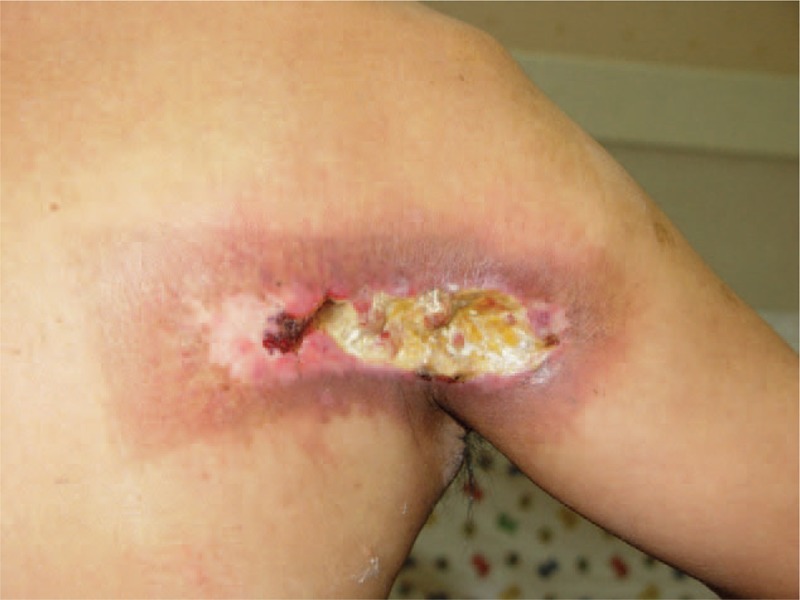
A fully developed cardiac fluoroscopy-induced radiation ulcer. A 60-year-old man had a painful deep large ulcer surrounded by a well-demarcated sclerotic dyspigmented patch on right subscapular and arm for 2 months.

Although all of lesions eventually became large refractory ulcers, the early presentation were mild forms of radiation skin damage, that is, radiation dermatitis without ulcer, in all cases. The most common early manifestation was a concentric target-like lesion with central erosion, peripheral white sclerotic zone and an outer hyperpigmented zone similar to flame burn injuries (Fig. 1). The square shape and the distribution of color perfectly reflected the exposure field of fluoroscopic radiation and underlying radiation-absorbed dose. On the other hand, the initial skin sign could mimic either allergic contact dermatitis presenting as a well-demarcated erythematous patch (Fig. 2), or scleroderma presenting as an atrophic dyspigmented plaque (Fig. 3). Despite of receiving regular wound care, including application of topical antibiotic ointments, conventional wound dressing, and use of hydrocolloid dressing, these lesions all progressed to deep necrotic ulcers with or without secondary infection (Fig. 4). Based on clinical inspection at first presentation to clinic, only 1 case had been ever correctly diagnosed before the skin ulcer became refractory.

Radiation ulcer is located in where the radiation beam enters. During performing percutaneous coronary angiography, it requires different radiator angles by operating radiator to visualize different coronary arteries. Among these 9 patients, the location of radiation ulcer was in the right subscapular region in 8 cases with or without involvement of auxiliary and arm area. That area is the common site where is the radiation beam entrance site for visualizing right coronary artery. Only 1 case had lesions in his mid back.

**FIGURE 5:**
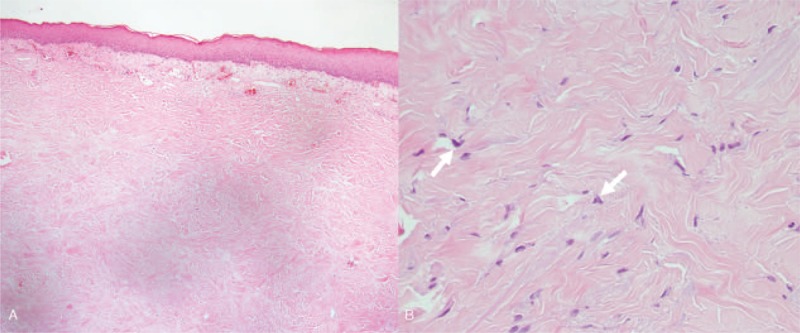
Histopathology of cardiac fluoroscopy induced skin damage. (A) Histopathology showed a patchy lymphatic infiltration and dilated vessels in the superficial dermis. In the mid and lower dermis, there is a sclerotic background composed of haphazardly arranged thick collagen fibers, absence of adnexal structures, and scattered atypical fibroblasts in the reticular dermis. These features are consistent with the diagnosis of radiation dermatitis. However, scleroderma may present the similar changes. (B) Nevertheless, the atypical fibroblasts (arrow) were conspicuously seen in some foci. That was the feature of radiation skin damage, not of scleroderma.

Histopathology studies were performed in 6 cases, while the clinicians and pathologists were not aware of patients’ radiation exposure history. Although all the histopathology studies showed features compatible with radiation skin damage, including absence of adnexal structures, sclerosis of reticular dermis, and presence of atypical stellate-shaped fibroblasts (Fig. 5), only 1 case had been diagnosed correctly as radiation dermatitis. To make thing worse, skin biopsy exacerbated the preexisting radiation dermatitis.

**FIGURE 6:**
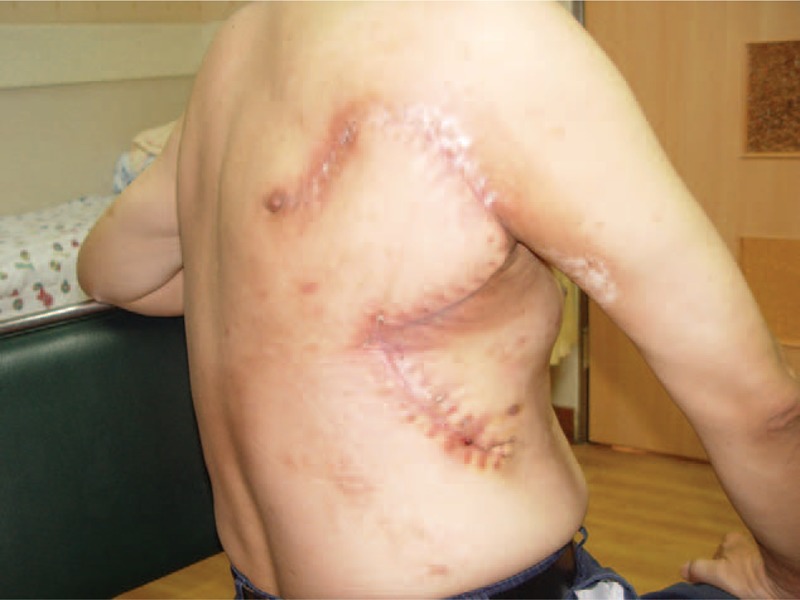
Outcome of surgical intervention for cardiac fluoroscopy-induced radiation ulcer. A 52-year-old man had radical excision of radiation ulcer and reconstruction of rotation flap. One month after the operation, good healing was noted.

All medical managements to these wounds failed to promote healing, including conventional wound care, hyperbaric oxygen therapy, hydrocolloid dressing, artificial biologic coverage, and conservative wound debridement. Eight patients (8/9) eventually received surgical treatments. Surgical intervention including radical wound debridement and reconstruction were arranged according to the patients’ condition. These managements eventually brought complete wound healing in each and every patients (Fig. 6), although their treatment courses often were long and complicated involving repeated excisions and wound closures.

## DISCUSSION

We presented 9 patients having radiation ulcers, which were identified among the individuals who received cardiac angiographic intervention during 2012 and 2013 in our hospital. This represents the incidence of radiation ulcer is 0.34% per practice (9/2570) or 0.42% per patient (9/2124). In this study, we used strict inclusion criteria to identify the cases with radiation ulcer; that is, only patients, whose clinical photos were available for reviewing, would be included for further evaluation. However, pictures were not routinely taken for every patient in the clinic of dermatology or plastic surgery. Besides radiation ulceration following PCI usually developed a period of time (week or months) after the cardiac procedure and the initial presentation of this complication is mild. Thus some of the patients could not relate current skin problem with previous PCI procedures and might seek medical help as their conveniences, not the original hospital. Therefore, it is highly possible that our study underestimates the exact incidence of radiation ulcer. Despite of the aforementioned limitations, the incidence rate in our report is still much higher than that of any other reports before.^8^

Accurately measuring the radiation absorption dose at the skin entrance site is difficult. Therefore, the exact radiation exposure dose can only be estimated indirectly by using procedure time and fluoroscopy time. During the normal mode of fluoroscopy for regular coronary angiography, patient is usually exposed to a radiation dose at a rate around 0.02 to 0.05 Gy/min. In general, the mean duration of the each procedure is between 1 and 2 h, thus the cumulative radiation dose is theoretically at a maximum of 3 Gy in an individual PCI.^9^ However, in reality, the radiation dose associated with fluoroscopic procedures is dependent on multiple factors. For example, a higher radiation emission rate (up to 0.2 Gy/min) will occur automatically decided by computer setting when operators require higher image resolution or when the radiation needs to pass a longer distance through human body. Therefore, the actual cumulative radiation dose is usually underestimated, if we use only the procedure time (or fluoroscopy time) to calculate it.

Chronic radiation skin damages, which may not be preceded by acute radiation dermatitis, develop weeks to months after radiation exposure with refractory symptoms such as pruritus and pain. Skin ulcer can be triggered and worsening by minor trauma caused by scratching, applying topical agents or hot packing employed by patients to relieve the associated pruritus and pain. Contact dermatitis, fixed drug eruption and scleroderma are the possible clinical differential diagnoses. But the typical location on back, bizarre shape with very sharp margin, and concentrically colored distribution of the lesion are characteristic to radiation dermatitis. These features help remind clinicians to ask patients about any history of previous cardiac catheter intervention. Although the cause-and-effect relationship between radiation and the cutaneous presentation seems obvious once it is diagnosed, a timely identification of fluoroscopy-induced chronic radiation damage is often challenging. The difficulties in making the correct diagnosis are usually owing to the variable onset interval, misleading concomitant symptoms, and lack of awareness of fluoroscopy-induced radiation skin damage.

On the other hand, radiation induced-morphea (RIM) is a possible differential diagnosis in the literature.^10^ RIM rarely leads to nonhealing ulceration and it potentially extends exceeding outside the radiation exposure field. It mainly appears in the female patients in the literature and has been reported that systemic sclerosis is a relative risk factor for developing an exaggerated postirradiation fibrosis. Unlike to the characters of RIM, all of our patients were male, and had severe refractory painful ulcers confined on the original radiation exposure area. And none of them had history of systemic sclerosis or any other autoimmune disease. These features favor the diagnosis of radiation ulcer. Therefore, these cases were diagnosed with radiation ulcer, not RIM.

The histological features of these lesions include epidermal atrophy, dermal sclerosis (eosinophilic homogenized sclerosis of dermal collagen), dilated superficial blood vessels, loss of adnexal structures (hair follicle and sweat duct), and increased atypical stellate-shaped fibroblasts.^11,12^ In most situations, the proper diagnosis of radiation skin damage can be made by combining the clinical presentations and a radiation exposure history. Skin biopsy should be reserved when histology pictures are needed for a correct diagnosis such as radiation malignancy or invasive deep infections are suspected. This is because radiation ulcers’ histological features are not characteristic, and scleroderma and lichen sclerosis may present similar pathologic findings. To make thing worse, an incision biopsy creates new wound and potentially exacerbates the preexisting damaged skin and ulcer.

Up to now, there is no consensus or guidelines for managing radiation ulcer. Generally speaking, conservative treatments may be effective for radiation dermatitis (without ulcer). These managements include appropriate skin protection and avoidance of unnecessary surgical procedures. However, once radiation ulcer occurs, surgical intervention becomes necessary to promote wound healing.^13,14^ In our experience, aggressive radical excision following by reconstruction with local flap is effective in treating radiation ulcers with refractory course.

To prevent this complication, minimizing the radiation dose is the cornerstone and this is possible by following current regulations and several new approaches.^15–17^ For example, by simply adjusting preset standard frame rates for acquisition and fluoroscopy, as well as modifications and upgrades to the newer X-ray equipment, Sawdy et al^18^ achieved a significant reduction (66%) of total radiation exposure to patients. This is imperative not only for the patient but also for the medical staff because backscattered radiation can accumulate up to 25% to 40% of direct radiation.^19,20^ On the other hand, whenever a substantial radiation dose level has been reached, the patient should be informed and appropriately educated about skin care. Regular dermatology monitoring and evaluation are necessary in these patients.^15,16^ This is both for surveying radiation dermatitis and radiation malignancy. Malignancies arising from chronic radiation dermatitis have been documented in other types of radiation exposure, such as radiation therapy for cancer or benign disease. Squamous cell carcinoma, basal cell carcinoma, and sarcoma are the most common types of malignancies.^21,22^ It was reported that 0.9% of cancers in the United States were caused by diagnostic X-rays.^23^ Therefore, it is sensible to speculate that the cardiac angiographic intervention might pose an even higher risk of malignancy,^24^ giving its higher radiation exposure.

In conclusion, radiation skin damage is an overlooked complication after wide spread application of cardiac angiographic interventions. Prolonged procedure time, accumulative multiple procedures, RCA with CTO, obesity, and diabetes are frequent characteristics among the patients of cardiac fluoroscopy induced radiation ulcer in this study. To minimize the incidence and severity of radiation skin injuries, medical attention is required before, during, and after the procedure. Postintervention regular dermatology monitoring and patient education about skin care are pivotal not only for the radiation dermatitis but also for the possible malignancy. When facing a sharply demarcated patch with or without ulcer on the back in shape of rectangle or square, physicians should be alert to the possibility of radiation dermatitis. Skin biopsy should be avoided if the clinical presentation and history of radiation exposure are typical. Radical excision with local flap is an effective treatment for recalcitrant radiation ulcers.

## References

[1] Aerts A Decraene T van den Oord JJ Chronic radiodermatitis following percutaneous coronary interventions: a report of two cases. *J Eur Acad Dermatol Venereol* 2003; 17:340–343. 12702082 10.1046/j.1468-3083.2003.00687.x

[2] Herz-Ruelas ME Gomez-Flores M Moxica-Del Angel J Ulcerated radiodermatitis induced after fluoroscopically guided stent implantation angioplasty. *Case Rep Dermatol Med* 2014; 2014:3. 10.1155/2014/768624 PMC4168146 25276441

[3] Khouzam RN Soufi MK Nakhla R Outpatient percutaneous coronary intervention: has its time come? *J Invasive Cardiol* 2014; 26:E167–E169. 25481000

[4] Mozaffarian D Benjamin EJ Go AS Heart disease and stroke statistics—2015 update: a report from the American Heart Association. *Circulation* 2015; 131:e29–e322. 25520374 10.1161/CIR.0000000000000152

[5] Brilakis E Manual of Coronary Chronic Total Occlusion Interventions: A Step-by-Step Approach. Waltham, MA: Elsevier; 2013.

[6] Fetterly KA Lennon RJ Bell MR Clinical determinants of radiation dose in percutaneous coronary interventional procedures: influence of patient size, procedure complexity, and performing physician. *JACC Cardiovasc Interv* 2011; 4:336–343. 21435613 10.1016/j.jcin.2010.10.014

[7] Michael TT Karmpaliotis D Brilakis ES Temporal trends of fluoroscopy time and contrast utilization in coronary chronic total occlusion revascularization: insights from a multicenter united states registry. *Catheter Cardiovasc Interv* 2015; 85:393–399. 24407867 10.1002/ccd.25359 PMC4090298

[8] Abdelaal E Plourde G MacHaalany J Effectiveness of low rate fluoroscopy at reducing operator and patient radiation dose during transradial coronary angiography and interventions. *JACC Cardiovasc Interv* 2014; 7:567–574. 24746649 10.1016/j.jcin.2014.02.005

[9] Miller DL Balter S Noonan PT Minimizing radiation-induced skin injury in interventional radiology procedures. *Radiology* 2002; 225:329–336. 12409563 10.1148/radiol.2252011414

[10] Chan J-Y Chu C-Y Chronic radiodermatitis following percutaneous coronary interventions. *Dermatol Sin* 2004; 22:148–152.

[11] Boncher J Bergfeld WF Fluoroscopy-induced chronic radiation dermatitis: a report of two additional cases and a brief review of the literature. *J Cutan Pathol* 2012; 39:63–67. 21752059 10.1111/j.1600-0560.2011.01754.x

[12] Spiker A Zinn Z Carter WH Fluoroscopy-induced chronic radiation dermatitis. *Am J Cardiol* 2012; 110:1861–1863. 22980965 10.1016/j.amjcard.2012.08.023

[13] Nishimoto S Fukuda K Kawai K Supplementation of bone marrow aspirate-derived platelet-rich plasma for treating radiation-induced ulcer after cardiac fluoroscopic procedures: a preliminary report. *Indian J Plast Surg* 2012; 45:109–114. 22754164 10.4103/0970-0358.96599 PMC3385373

[14] Otterburn D Losken A Iatrogenic fluoroscopy injury to the skin. *Ann Plast Surg* 2010; 65:462–465. 20948414 10.1097/SAP.0b013e3181d6e2d3

[15] Chambers CE Fetterly KA Holzer R Radiation safety program for the cardiac catheterization laboratory. *Catheter Cardiovasc Interv* 2011; 77:546–556. 21254324 10.1002/ccd.22867

[16] Miller DL Balter S Schueler BA Clinical radiation management for fluoroscopically guided interventional procedures. *Radiology* 2010; 257:321–332. 20959547 10.1148/radiol.10091269

[17] Hirshfeld JW Jr Balter S Brinker JA ACCF/AHA/HRS/SCAI clinical competence statement on physician knowledge to optimize patient safety and image quality in fluoroscopically guided invasive cardiovascular procedures: a report of the American College of Cardiology Foundation/American Heart Association/American College of Physicians Task Force on Clinical Competence and Training. *Circulation* 2005; 111:511–532. 15687141 10.1161/01.CIR.0000157946.29224.5D

[18] Sawdy JM Kempton TM Olshove V Use of a dose-dependent follow-up protocol and mechanisms to reduce patients and staff radiation exposure in congenital and structural interventions. *Catheter Cardiovasc Interv* 2011; 78:136–142. 21681901 10.1002/ccd.23008

[19] Balter S Hopewell JW Miller DL Fluoroscopically guided interventional procedures: a review of radiation effects on patients’ skin and hair. *Radiology* 2010; 254:326–341. 20093507 10.1148/radiol.2542082312

[20] Martin CJ Measurement of patient entrance surface dose rates for fluoroscopic X-ray units. *Phys Med Biol* 1995; 40:823–834. 7652010 10.1088/0031-9155/40/5/008

[21] Schwartz RA Burgess GH Milgrom H Breast carcinoma and basal cell epithelioma after X-ray therapy for hirsutism. *Cancer* 1979; 44:1601–1605. 498032 10.1002/1097-0142(197911)44:5<1601::aid-cncr2820440510>3.0.co;2-8

[22] Miller AB Howe GR Sherman GJ Mortality from breast cancer after irradiation during fluoroscopic examinations in patients being treated for tuberculosis. *N Engl J Med* 1989; 321:1285–1289. 2797101 10.1056/NEJM198911093211902

[23] de Gonzalez AB Darby S Risk of cancer from diagnostic X-rays: estimates for the UK and 14 other countries. *Lancet* 2004; 363:345–351. 15070562 10.1016/S0140-6736(04)15433-0

[24] Hung MC Hwang JJ Cancer risk from medical radiation procedures for coronary artery disease: a nationwide population-based cohort study. *Asian Pac J Cancer Prev* 2013; 14:2783–2787. 23803032 10.7314/apjcp.2013.14.5.2783

